# MCD diet-induced steatohepatitis generates a diurnal rhythm of associated biomarkers and worsens liver injury in *Klf10* deficient mice

**DOI:** 10.1038/s41598-020-69085-w

**Published:** 2020-07-22

**Authors:** Pierre S. Leclère, Déborah Rousseau, Stéphanie Patouraux, Sophie Guérin, Stéphanie Bonnafous, Aline Gréchez-Cassiau, Anthony A. Ruberto, Carmelo Luci, Malayannan Subramaniam, Albert Tran, Franck Delaunay, Philippe Gual, Michèle Teboul

**Affiliations:** 1 Université Côte D’Azur, CNRS, INSERM, iBV, Nice, France; 2 Université Côte D’Azur, INSERM, C3M, Nice, France; 3 Université Côte D’Azur, CHU, INSERM, C3M, Nice, France; 4 Department of Biochemistry and Molecular Biology, Mayo Clinic, Rochester, MN USA

**Keywords:** Physiology, Metabolism

## Abstract

A large number of hepatic functions are regulated by the circadian clock and recent evidence suggests that clock disruption could be a risk factor for liver complications. The circadian transcription factor Krüppel like factor 10 (KLF10) has been involved in liver metabolism as well as cellular inflammatory and death pathways. Here, we show that hepatic steatosis and inflammation display diurnal rhythmicity in mice developing steatohepatitis upon feeding with a methionine and choline deficient diet (MCDD). Core clock gene mRNA oscillations remained mostly unaffected but rhythmic *Klf10* expression was abolished in this model. We further show that *Klf10* deficient mice display enhanced liver injury and fibrosis priming upon MCDD challenge. Silencing *Klf10* also sensitized primary hepatocytes to apoptosis along with increased caspase 3 activation in response to TNFα. This data suggests that MCDD induced steatohepatitis barely affects the core clock mechanism but leads to a reprogramming of circadian gene expression in the liver in analogy to what is observed in other experimental disease paradigms. We further identify KLF10 as a component of this transcriptional reprogramming and a novel hepato-protective factor.

## Introduction

Non-alcoholic fatty liver diseases (NAFLDs) are the most frequent chronic liver pathologies worldwide with a global prevalence ranging from 22 to 28%^1^. NAFLDs cover a spectrum of hepatic abnormalities ranging from reversible steatosis to non-alcoholic steatohepatitis (NASH) and steatofibrosis which may lead to cirrhosis and ultimately hepatocellular carcinoma^2^. NASH is characterized by (i) fatty hepatocytes, (ii) inflammatory foci, (iii) ballooned hepatocytes and (iv) increased hepatocytes apoptosis and necrosis. Liver injury associated with NASH leads to elevated systemic levels of alanine transaminase (ALT) and aspartate transaminase (AST) activity^3^. Sustained activation of cellular stress in lipid-overloaded hepatocytes and increased activation of death receptor signaling including the Tumor Necrosis Factor alpha (TNFα) pathway, have been associated with hepatocellular injury during NASH^4^. Additional modes of cell death including necroptosis and pyroptosis could also participate to liver injury during NASH^5,6^. Hepatocyte injury derived signals such as Danger Associated Molecular Pattern Signals (DAMPs) as well as pro-inflammatory cytokines and chemokines are also strong drivers of NASH progression^7^. Although an increasing number of clinical trials are now in progress, approved pharmacological therapy for burned-out NASH is still missing.

The mammalian circadian timing system (CTS) coordinates most of physiology and behavior with the external light/dark cycle. This system is organized hierarchically with a central pacemaker in the suprachiasmatic nuclei (SCN) of the hypothalamus that receives light inputs from the retina and in turn entrains cellular clocks present in virtually all peripheral tissues and extra-hypothalamic brain regions via internal synchronizers^8^. Cellular circadian clocks control a vast array of biological processes in a tissue specific manner and the liver is the organ showing the most extensive circadian regulation^9^. Many features of modern society life style including sleep deprivation, shiftwork, light at night, overfeeding, and night eating contribute to the misalignment of internal body time with the environmental cycle and this may in turn compromise health^10^. Consistently, epidemiological studies increasingly support the notion that a poorly synchronized or disrupted CTS is an important risk factor for the development of many chronic metabolic diseases including dyslipidemia, overweight and insulin resistance, and cancer^11–13^. Reciprocally, circadian physiology can be altered by diseases^14–16^. This is exemplified by the reprogramming of the liver and serum metabolome as well as hepatic transcriptome occurring in obese mice^17–20^. The interaction of the circadian clock with several mechanisms involved in the onset of hepatic-steatosis and its progression to NASH has been well documented in several reviews^21–23^. Global and tissue specific knock-out or mutation of clock genes (*Rev-erbα/β*^*-/-*^*, Cry1/2*^*-/-*^*, Bmal1*^*-/-*^* and ClockΔ19, Bmal1*^*Δhep*^) promote hepatic steatosis, and alter blood glucose and lipid homeostasis in lean and high fat diet (HFD) fed obese mice^24–28^*.* Accordingly, sustained chrono-disruption in mice via experimental chronic jet lag leads to spontaneous development of NASH and hepatocellular carcinoma^23,29^. Several transcription factors belonging to the *Krüppel Like Factor* (*KLF*) family have recently emerged as important circadian regulators involved in liver functions and diseases^30–32^. While some of these *KLF* family members including KLF4 and KLF6 are potential players in NAFLD development^33–35^, the significance of KLF10 in liver physiopathology remains unknown although it has been involved in several mechanisms which are dysregulated in NAFLD^36–38^. We previously reported that hepatic *Klf10* is a circadian output of the clock as a result of a direct regulation by the BMAL1/CLOCK heterodimer. Transcriptome profiling of *Klf10*^*-/-*^ mouse liver further identified a differentially expressed gene set that was significantly enriched for lipid and carbohydrate metabolism processes. In addition, KLF10 was shown to regulate hepatic gluconeogenesis through the direct transcriptional repression of *Pepck*^30^. It has also been recently reported that *Klf10* hepatic expression is strongly increased in fatty liver of obese mice^39^. KLF10 has also been shown to be involved in inflammation and cell death processes^37,40^. All these features suggest that KLF10 may have a role in the development of steatohepatitis that remains to be explored. In the current study, we use mice fed a methionine and choline deficient diet (MCDD), which is a rodent model of oxidative-stress mediated steatohepatitis. On the one hand, a lack of choline in this diet, hampers the export of triglycerides (TG) via a very low-density lipoprotein (VLDL) packaging from hepatocytes, resulting in hepatic steatosis. On the other hand, the essential amino acid methionine is required for the synthesis of *S*-adenosylmethionine (SAM) and glutathione, which are both antioxidants. Although this model was widely used and despite the increasing numbers of reports linking circadian disorders and NAFLD, no study investigated the influence of the MCDD challenge on the circadian rhythmicity. In the present study, we examine the steatohepatitis features around the 24-h cycle and the role of the circadian transcription factor KLF10 in the progression of the disease, in mice fed a MCDD.

## Results

### Diurnal rhythmicity of liver steatosis, inflammation and fibrosis priming in MCDD fed mice

**Figure 1:**
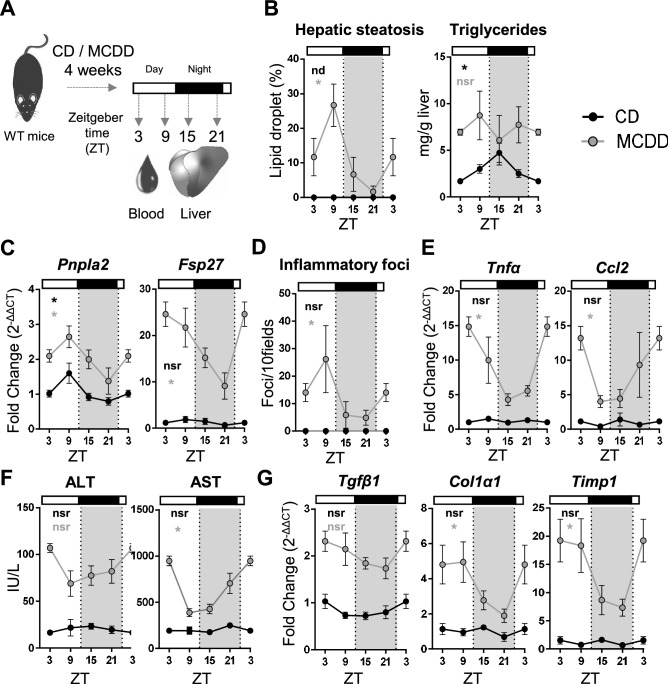
Hepatic steatosis and inflammation display circadian rhythmicity during steatohepatitis. (**A**) WT male mice were fed on a control diet (CD) (n = 3–4 mice per ZT) or a methionine and choline deficient diet (MCDD) (n = 5–6 mice per ZT) for 4 weeks and the blood and liver were sampled around the clock every 6 h. (**B**) Quantification of hepatic steatosis from H&E stained liver sections (%) and total liver triglyceride contents (mg/g of tissue). (**C**) Hepatic gene expression of *Pnpla2* and *Fsp27*. (**D**) quantification of inflammatory foci from the H&E staining of liver tissue section samples. (**E**) Gene expression of *Tnfα* and *Ccl2*. (**F**) Serum ALT and AST activity (IU/L). **(G)** Gene expression of *Tgfβ, Col1α1 and Timp1.* All data are expressed as mean ± SEM. Gene expressions are normalized to *B2m* and expressed relative to the CD ZT3 level. Rhythmicity of the liver complications and related gene expressions was evaluated by nonlinear regression cosine fitting analysis (cosinor) (Supplementary Table S1). The rhythmicity of each parameter is indicated on their respective graph with the corresponding color. ZT3 values were double-plotted to complete the 24 h cycle. *, rhythmic (p < 0.05), nsr, non-significantly rhythmic, nd, not detected.

Several experimental paradigms using nutritional challenges in mice have shown a reprogramming of the circadian transcriptome and physiology in many organs suggesting that these pathogenic conditions have the potential to unveil disease related rhythmic patterns^15,17–20^. To evaluate whether steatohepatitis features, including hepatic steatosis, inflammation and liver injury, display a circadian rhythmicity, WT mice were fed with a control diet (CD) or a methionine and choline deficient diet (MCDD) for 4 weeks. Blood and liver were collected every 6 h over 24 h at ZT3, 9, 15 and 21, ZT0 and ZT12 being the time when the light is switched on and off, respectively (Fig. 1A). Hepatic steatosis evaluated by the percentage of lipid droplets displayed a robust diurnal rhythmicity with a calculated acrophase during the rest phase at ZT8 (Fig. 1B, Supplementary Table S1) upon MCDD challenge**.** Consistent with this observation, hepatic expression of *Fsp27* (also known as *Cidec*) and *Pnpla2* which encode for two proteins involved in lipid droplet fusion and unpacking respectively, also showed a rhythmic profile with a peak of expression during the rest phase (Fig. 1C, Supplementary Table S1). The hepatic triglyceride (TG) content displayed rhythmicity in CD fed mice as expected from previous work^41^. Expectedly, the TG content was increased upon MCDD, but was no longer rhythmic (Fig. 1B, Supplementary Table S1). These observations suggest that although the elevated content of TG remains constant during the 24 h cycle, the dynamics of CIDE-regulated lipid droplet formation may be a rhythmic process. Hepatic inflammation evaluated by the number of inflammatory foci also exhibited a robust diurnal rhythmicity during steatohepatitis (Fig. 1D, Supplementary Table S1). Interestingly, expression of the inflammatory markers *Tnfα* and *Ccl2*, which are not rhythmic in the CD group, displayed a de novo rhythmicity upon MCDD-induced steatohepatitis (Fig. 1E, Supplementary Table S1). As expected, upon MCDD, both AST and ALT activities were higher in the MCDD fed mice than in the CD fed mice (Fig. 1F, Supplementary Table S1). We observed that both ALT and AST activity varied depending on the time of the day, ALT activity being at ZT3 than at ZT9, and AST activity displaying a 24 h oscillation. (Fig. 1F, Supplementary Table S1). In addition, the fibrosis priming markers *Tgfβ1, Col1α1 and Timp1* were not rhythmic upon CD and, as expected, were upregulated upon MCDD*.* While *Tgfβ1* did not display circadian rhythmicity, *Col1α1* and *Timp1* both gained rhythmicity upon MCDD (Fig. 1G, Supplementary Table S1). Collectively these results demonstrate that multiple markers that have an arrhythmic expression in chow diet display an up-regulation associated with a time-of-day dependent variation in the MCDD induced NASH mouse model.

### Steatohepatitis induces discrete alterations of the liver molecular clock and abolishes *Klf10* oscillation

**Figure 2:**
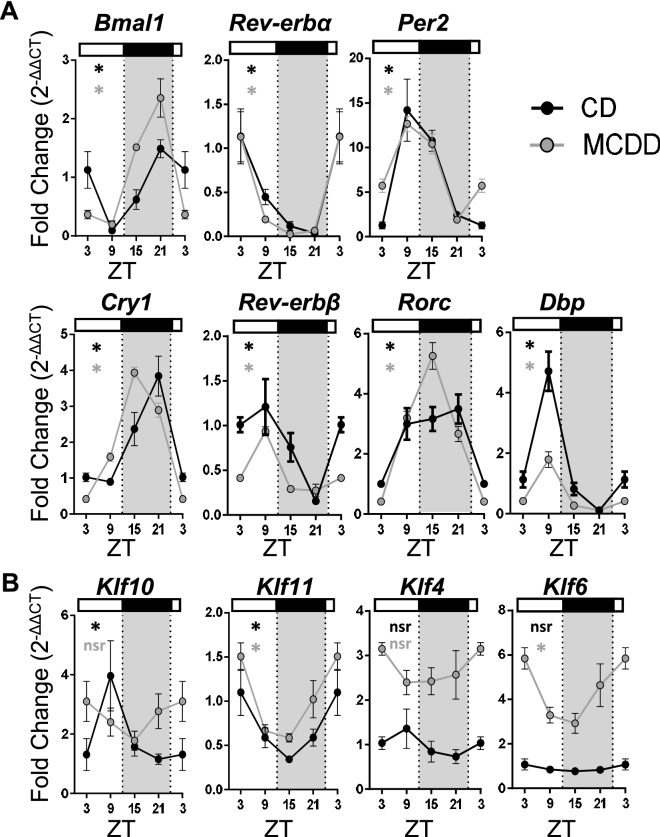
Steatohepatitis in mice leads to *Klf10* loss of rhythmicity in the liver. WT male mice were fed on a control diet (CD) (n = 3–4 mice per ZT) or a methionine and choline deficient diet (MCDD) (n = 5–6 mice per ZT) for 4 weeks and the blood and liver were sampled around the clock every 6 h. (**A)** Gene expression of *Bmal1, Rev-erbα, Per2, Cry1, Rev-erb-β, Rorc* and *Dbp* in mice fed a CD or a MCDD*.* (**B**) Gene expression of *Klf10*, *Klf11*, *Klf4* and *Klf6.* All data are expressed as mean ± SEM. Gene expressions are normalized to *B2m* and expressed relative to the CD ZT3 level. Rhythmicity of the hepatic gene expressions was evaluated by nonlinear regression cosine fitting analysis (cosinor) (Supplementary Table S2). The rhythmicity of each parameter is indicated on their respective graph with the corresponding color. ZT3 values were double-plotted to complete the 24 h cycle. *, rhythmic (p < 0.05), nsr, non-significantly rhythmic, nd, not detected.

**Figure 3:**
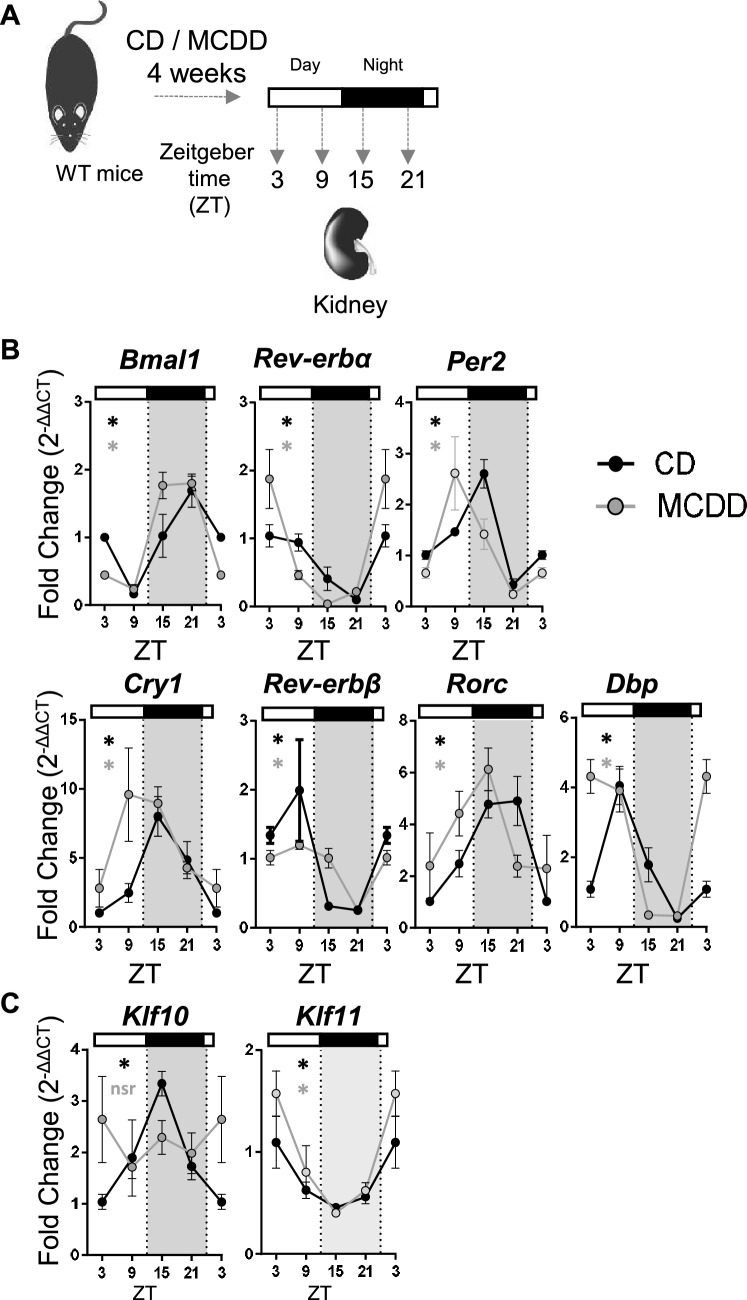
Steatohepatitis in mice leads to *Klf10* loss of rhythmicity in the kidney. (**A)** WT male mice were fed on a control diet (CD) (n = 2–4 mice per ZT) or a methionine and choline deficient diet (MCDD) (n = 5–6 mice per ZT) for 4 weeks and kidneys were sampled around the clock every 6 h. (**B)** Gene expression of *Bmal1, Rev-erbα, Per2, Cry1, Rev-erb-β, Rorc* and *Dbp.*(**C**) Gene expression of *Klf10* and *Klf11.* All data are expressed as mean ± SEM. Gene expressions are normalized to *B2m* and expressed relative to the CD ZT3 level. Rhythmicity of the renal gene expressions was evaluated by nonlinear regression cosine fitting analysis (cosinor). (Supplementary Table S3). The rhythmicity of each parameter is indicated on their respective graph with the corresponding color. ZT3 values were double-plotted to complete the 24 h cycle. *, rhythmic (p < 0.05), nsr, non-significantly rhythmic, nd, not detected.

To investigate whether the observed de novo rhythmicity of steatohepatitis features could be linked to a modification of the hepatic circadian clock gene network, we profiled clock and clock-controlled gene expression in the same set of mice. We observed robust oscillations in both normal and pathogenic livers for all analyzed core clock genes including *Bmal1*, *Rev-erbα* and *β*, *Per2*, *Cry1*, *Rorc* (Fig. 2A, Supplementary Table S2). However, we noticed that the phase of *Bmal1 and Cry1* was advanced by approximately 3 h and that amplitude of *Rorc* was augmented by twofolds (Fig. 2A, Supplementary Table S2). In contrast the profile of the clock-controlled transcription factor *Dbp* was more severely altered as its amplitude was dampened by twofolds in MCDD fed mice (Fig. 2A). We also found that *Klf10* expression became arrhythmic upon this challenge (Fig. 2B, Supplementary Table S2). To address whether the observed changes in clock and clock-controlled gene expression are restricted to the liver, we also determined the expression of the same set of genes in the kidney as it has been described that steatohepatitis and development of chronic kidney disease are linked^42^ (Fig. 3A). Interestingly, clock genes were also rhythmic in both groups of mice with discrete modifications of their phase and amplitude (Fig. 3B, Supplementary Table S3). However, among all the evaluated genes, *Klf10* was also the only gene becoming arrhythmic upon MCDD in the kidney (Fig. 3C, Supplementary Table S3). We next determined whether this loss of rhythmicity was also observed for other *Klfs* known to be linked to liver physiology and disease*.* As shown in Fig. 2B and Supplementary Table S2, hepatic expression of *Klf4,* previously described in the M2 polarization of Kupffer cells during NASH^34^, was increased upon MCDD. Similarly, *Klf6,* which was associated with NAFLD and fibrosis^33,43^, was also up regulated and displayed a rhythmic pattern. In contrast, expression of the *Klf10* paralog *Klf11,* which has been previously reported to be important in hepatic lipid metabolism^35^, was not affected with the development of steatohepatitis in liver. Collectively, these gene expression profiles suggest that the MCDD challenge has no dramatic effects on the core circadian clock dynamics. Conversely the clock output *Klf10* appears to be depending on MCDD induced pathways that overcome its physiological circadian regulation normally occurring in healthy organs.

### Lack of KLF10 does not impact on the development of MCDD induced steatosis and inflammation

**Figure 4:**
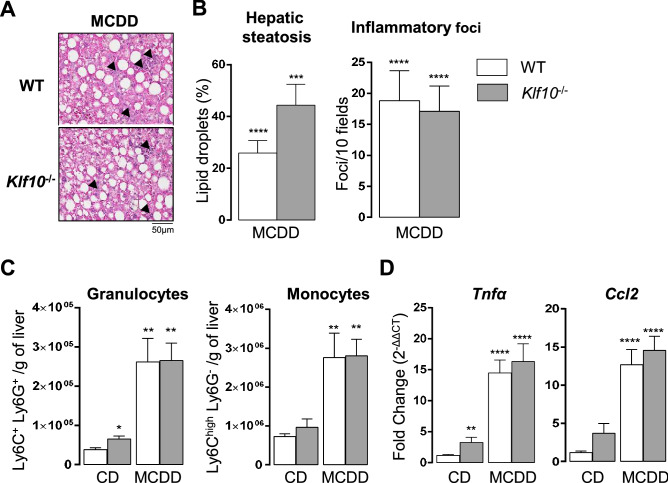
*Klf10* deficiency does not impact the development of hepatic steatosis and inflammation during steatohepatitis in mice. WT and *Klf10 *^*-/-*^ male mice were fed on a methionine and choline deficient diet (MCDD) for 4 weeks and sacrificed at ZT 3. (**A**) Representative images, showing the presence of lipid droplets and inflammatory foci (black arrows), from H&E stained liver sections of WT and *Klf10*^-/-^ upon MCDD. (**B**) Quantification of hepatic steatosis and inflammatory foci from H&E stained liver sections (n = 8–11 mice/group). (**C**) Number of hepatic granulocytes and monocytes assessed by flow cytometry on hepatic non parenchymal cells stained for CD45, Ly6C and Ly6G. Data represents the number of cells/g of tissue (n = 5–6 mice/group). (**D**) Hepatic expression of *Tnfα* and *Ccl2* (n = 8–12 mice/group). All data are expressed as mean ± SEM. The mRNA levels are normalized to *B2m* and expressed relative the CD ZT3 level (n = 12). Statistically significance was analyzed using the Mann–Whitney test *vs* control group WT CD, * p < 0.05, ** p < 0.01, **** p < 0.0001.

Since the hepatic circadian expression of *Klf10* was strongly impacted upon the MCDD challenge, we then determined its significance during steatohepatitis. Wild type and *Klf10*^*-/-*^ mice were fed with MCDD for 4 weeks and livers were sampled at two different ZTs during the rest phase as the steatosis and inflammatory foci are more important during this time window of the 24-h cycle (ZT3 and ZT9). Following MCDD challenge, the number of lipid droplets and inflammatory foci were similar in both genotypes at both ZTs (Fig. 4A,B, Supplementary Fig. S1a, b). In accordance with the quantification of the inflammatory foci, the recruitment of granulocytes (Ly6C^+^/Ly6G^+^) and inflammatory monocytes (Ly6C^high^) into the liver were also similar in both genotypes at ZT3 (Fig. 4C). In addition, the hepatic expression of inflammatory markers *Tnfα* and *Ccl2,* was also similar in both genotypes at both ZTs (Fig. 4D and Supplementary Fig. S1c). This data indicates that at this stage of the MCDD induced steatohepatitis, KLF10 does not appear to play a key role in the development of hepatic steatosis and inflammation.

### Lack of KLF10 aggravates MCDD induced liver injury and TNFα induced hepatocyte death

**Figure 5:**
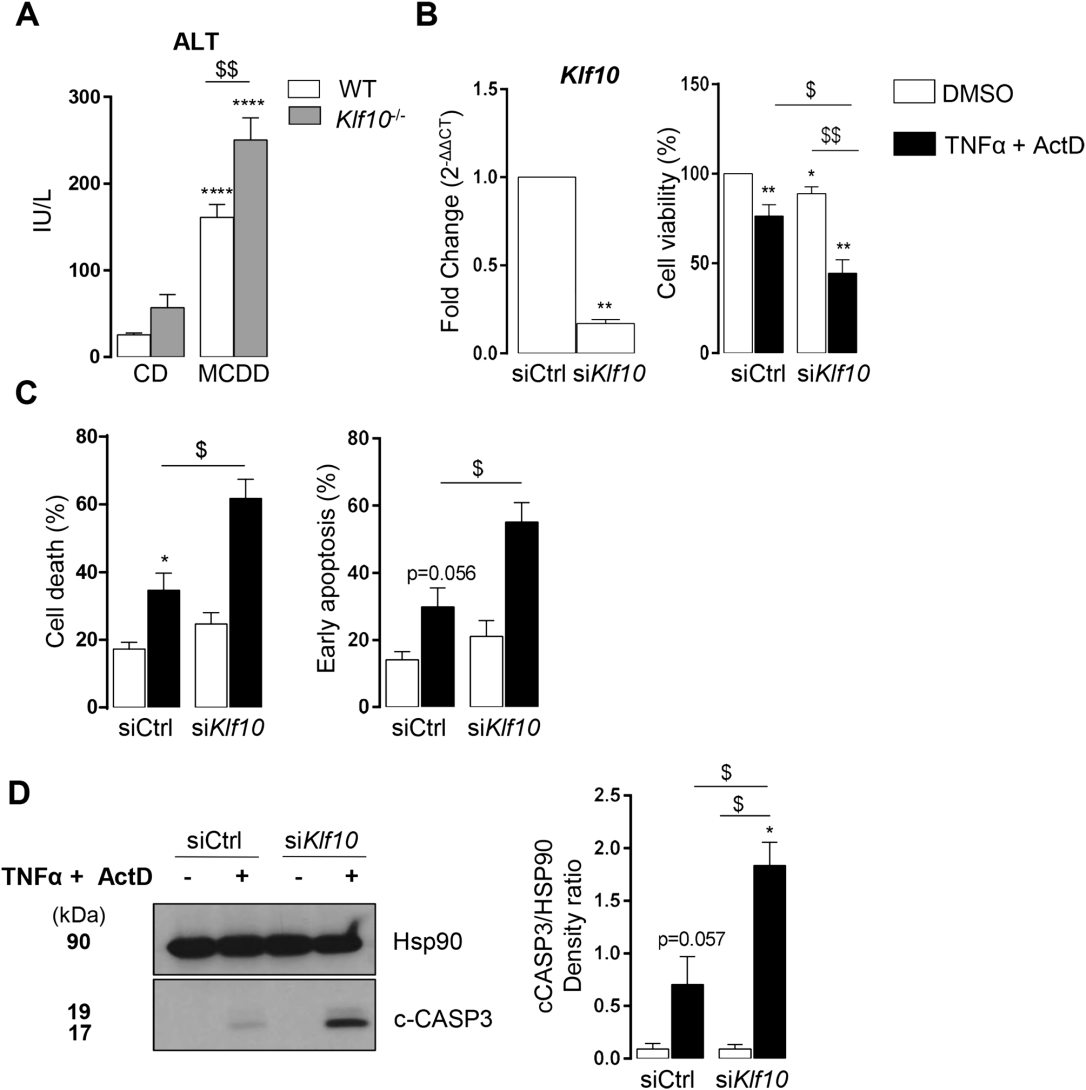
*Klf10* deficiency aggravates liver injury during steatohepatitis in mice and hepatocyte death in vitro. (**A**) Serum ALT activity of WT and *Klf10 *^*-/-*^ male mice were fed a methionine and choline deficient diet (MCDD) for 4 weeks and sacrificed at ZT 3 and 21 (n = 8–20 mice/group). (**B–D**) Mouse primary hepatocytes were transfected with control (siCtrl) or *Klf10* (si*Klf10*) siRNA and were stimulated with TNFα (20 ng/mL) and actinomycin D (0.1 µg/mL) for 16 h (**B–C**) or 12 h (**D**). **(B)** Hepatocyte *Klf10* gene expression (n = 6). Gene expression is normalized to *B2m* and expressed as relative expression of the untreated siCtrl condition, 72 h after transfection. Hepatocytes cell viability assessed by MTT assay (n = 6). **(C)** Hepatocyte cell death and apoptosis assessed by flow cytometry following 7aaD and annexinV staining (n = 5). **(D)** Western blot of cleaved CASPASE 3 fragment (17 kDa) (c-CASP3) and HSP90 protein (left) and densitometry quantification of cleaved CASPASE 3 was normalized to HSP90 in four independent experiments (right). All the data are expressed as mean ± SEM. Statistical significance was tested using the Mann–Whitney test; * and $ p < 0.05, ** and $$ p < 0.01,*** and $$$ p < 0.001, vs control group (**A**)/cells (**B,C,D**) (stars) or the indicated groups (**A**)/cells (**B, C, D**) (dollar symbols).

As liver injury is a hallmark of steatohepatitis, we compared serum ALT activity and liver TUNEL assay in WT and *Klf10*^*-/-*^ MCDD fed mice. ALT activity measurements were done in samples taken during the ZT21-ZT3 time window, as this corresponds to the highest activity (Fig. 1F). *Klf10*^*-/-*^ mice displayed significantly higher circulating ALT activity as compared to their WT controls suggesting that more hepatocyte death occurred in *Klf10*^*-/-*^ MCDD fed mice (Fig. 5A). In addition, liver sections of *Klf10*^*-/-*^ mice tended to contain more TUNEL positive nuclei than WT mice (Supplementary Fig. S2a) (evaluated at ZT3). Taken together, these results suggest that more hepatocyte death occurred in *Klf10*^*-/-*^ MCDD fed mice. To determine whether KLF10 could be a protective factor against hepatocyte death, we challenged *Klf10* silenced *vs* control mouse primary hepatocytes with TNFα and actinomycin D (ActD). TNFα is a well-known factor mediating hepatocyte death in steatohepatitis and is strongly upregulated in MCDD mice (Figs. 1E, 4D and Supplementary Fig. S1c). Unchallenged *Klf10*-silenced primary hepatocytes did not display any change regarding the expression of inflammatory markers (*Tnfα, Il6 and Ikb*) (Supplementary Fig. S2b). In response to TNFα/ActD, *Klf10*-silenced primary hepatocytes displayed reduced cell viability (Fig. 5B) associated with a higher percentage of dead cells and early apoptotic cells. (Fig. 5C). These cells also exhibited higher levels of activated (cleaved) caspase 3 (p17) in response to TNFα/ActD compared to control primary hepatocytes (Fig. 5D). These results suggest that hepatocytes lacking *Klf10* are more prone to apoptosis mediated by TNFα, which could support the aggravated liver injury observed in *Klf10*^*-/-*^ mice upon MCDD challenge.

### Lack of KLF10 enhanced the priming of hepatic fibrosis induced by 4 weeks MCDD challenge

**Figure 6:**
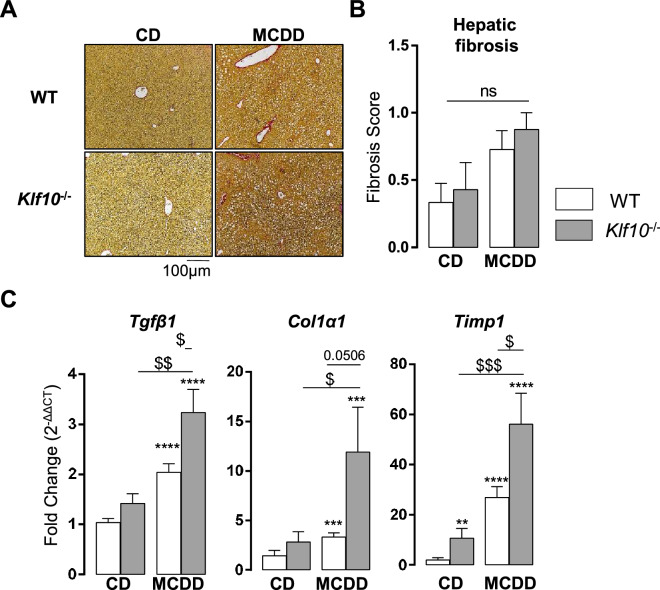
*Klf10* deficient mice display enhanced fibrosis priming upon MCDD challenge. (**A**) Representative images of liver sections sand tained with Picro Sirius red from WT and Klf10^-/-^ animals fed a CD or MCDD for 4 weeks (ZT3). (**B**) Quantification of hepatic fibrosis using the clinical fibrosis score (Ishak) (n = 7–12 mice/group). (**C**) Hepatic gene expression of *Tgfβ1, Col1α1*and *Timp1* in WT and *Klf10*^*-/-*^ mice sacrificed at ZT3 (n = 8–12 mice/group). All the data are expressed as mean ± SEM. The mRNA levels are normalized to *B2m* and expressed as relative to the CD level ZT3 (n = 12). Statistical significance was tested using the Mann-Whiney test vs the control group (stars) or the indicated groups (dollar symbols), **** p < 0.0001; ** and $$ p < 0.01; $ p < 0.05.

Since KLF10 deficiency was associated with increased liver injury, we then evaluated the hepatic fibrosis. Both genotypes developed no or few hepatic fibrosis after only 4 weeks MCDD feeding as assessed by Sirius red-stained liver sections (Fig. 6A,B). The absence of collagen deposition in WT mice was expected since we have previously reported that this requires a long lasting MCDD-challenge^44^. Interestingly, *Klf10*^*-/-*^ mice displayed significantly higher hepatic expression of fibrosis markers including *Tgfβ*, *Timp1* and *Col1α1* (Fig. 6C) compared to control animals**,** even before liver collagen accumulation. This suggests that these mice could be more prone to develop severe liver complications in response to long-lasting MCDD challenge by enhancing liver injury and fibrogenesis.

## Discussion

Recent studies have shown that nutritional challenges including high fat and ketogenic diets can reprogram extensively and in a tissue-specific manner the mouse circadian physiology^15,17–19^. This reprogramming is also observed in case of diseases such as rheumatoid arthritis^16^. In the present work we used the MCDD experimental paradigm to study the link between circadian timing and the development of steatohepatitis in the mouse. We found that the hepatic molecular clock is mostly resilient to this metabolic challenge and, that markers of steatosis and lobular inflammation, as well as serum transaminases activity displayed a diurnal rhythmicity. The finding that multiple challenges can consistently unveil pathology-related diurnal oscillations has several important basic mechanistic and translational implications.

The emergence of robust circadian rhythms in a pathologic setting may seem paradoxical at first glance, but this phenomenon is likely to reflect the fact that the CLOCK:BMAL1 heterodimer acts primarily as a pioneer factor regulating rhythmically chromatin accessibility to other transcription factors rather than being a necessary and sufficient driver of circadian transcription^45,46^. The extensive de novo rhythmicity induced by the high fat diet regimen was for example explained by the circadian nuclear accumulation of the lipogenic transcription factor PPARγ^18,20^. It is therefore conceivable that transcriptional responses to acute or chronic metabolic challenges involve genes which are normally repressed or not significantly expressed but which contain CLOCK:BMAL1 binding sites in their enhancers so that their pathogenic activation, by other transcription factors such as PPARγ, CREBH, NF-kB, gets temporally constrained by the circadian clock^18,47,48^. In line with this model, the *Fsp27/Cidec* mRNA coding for a protein promoting lipid droplet formation that serves as a marker of liver steatosis displayed a high amplitude rhythm with a phase preceding the peak of droplets in MCDD fed mice, and this gene was previously reported to be bound by the CLOCK:BMAL1 heterodimer despite being not expressed in healthy liver^46^ . Interestingly, the α and β isoforms of mouse *F*s*p27* are regulated by the peroxisome proliferator-activated receptor gamma (PPARγ) and Cyclic-AMP-responsive-element-binding protein H (CREBH) respectively^49,50^, which both become rhythmic. *Fsp27* could therefore become rhythmic in the in the MCDD model through a mechanism similar to that described by Murakami et al.^20^. An additional and not exclusive mechanism may also be attributed to a putative disruption of homeostatic microbiota rhythmicity by the MCDD model since this challenge was reported to alter the gut microbiota^51^. Importantly, disruption of homeostatic microbiome rhythmicity was reported to lead to genome-wide de novo oscillations in both intestine and liver^19,20,52,53^. Unexpectedly, the diurnal pattern of MCDD induced steatosis was not paralleled by a similar rhythm of hepatic TG. In high fat diet challenged mice, metabolomics revealed that hepatic glycerol levels are elevated and not rhythmic^17^. While the rhythm of intermediated products of fatty acid esterification and TG hydrolysis (DAG and MAG) have not been well established in fatty liver, our results could also suggest that lipid droplets dynamics rather than TG metabolism could be rhythmic in the MCDD hepatic steatosis model. The mechanism underlying such rhythmicity could operate at the level of droplet biogenesis and/or degradation, or alternatively be the result of interaction with clock-controlled cellular processes^54^ such as mitochondrial metabolism^55^, autophagy^56^ or the unfolded protein response^57,58^. One important role of lipid droplets besides the storage of TGs, steryl esters and retinyl esters, is to safely sequester otherwise toxic lipids such as overabundant non esterified fatty acids (NEFA). This protective function against lipotoxicity is probably the reason for the abundant accumulation of lipid droplets in many disease states such as NAFLD^59^. This implies that the intracellular toxicity of harmful lipids such as NEFAs is likely to be rhythmic.

Immune cells trafficking displays diurnal variations under basal conditions in multiple organs, including the liver^60,61^. Our data provides the evidence that there is a clear diurnal variation of inflammatory foci during the rest phase in the liver of mice fed a MCDD. Interestingly, this rhythmicity is close to what was observed in the aortic plaque during atherosclerosis^62^. This rhythm could be the result of a general inflammatory circadian homing code. Hepatic *Tnfα* and *Ccl2* gene expression gained rhythmicity with a peak of expression during the day (ZT2 and 4, respectively). TNFα and CCL2 play a crucial role in hepatic inflammation and leukocyte recruitment into the liver during steatohepatitis, respectively^63^. These observations are in line with previous studies in mouse models of inflammation mediated by *Plasmodium* infection and diet-mediated atherosclerosis^62,64^. In the latter, CCL2 was involved in the circadian recruitment of granulocytes and monocytes at the site of inflammation in a clock dependent fashion^62^.

In humans, NASH occurs in association with insulin resistance and obesity. In contrast, MCDD-fed mice lose weight, are hypoglycemic and do not have systemic insulin resistance, however they develop hepatic insulin resistance^65^. Our data obtained with the MCDD paradigm should stimulate further studies with models recapitulating metabolic disorders such as obesity and insulin resistance to investigate if the de novo oscillations we observe with the MCDD challenge are also features of NASH.

We previously showed that *Klf10* is a circadian clock-controlled gene that regulates metabolic genes in non-pathogenic liver^30^. Interestingly, this transcription factor loses rhythmicity with steatohepatitis. This could indicate that upon MCDD challenge, *Klf10* could be regulated by additional mechanisms independent of the CLOCK:BMAL1 heterodimer as previously described^30,66^. In addition, our data demonstrates that loss of KLF10 is associated with higher liver injury despite hepatic steatosis and inflammation similar to that seen in WT mice upon MCDD challenge. In accordance with the greater liver injury observed in *Klf10*^*-/-*^ mice, we show that these mutant mice display enhanced hepatic fibrosis priming, even before collagen accumulation. This indicates that KLF10 is required to attenuate hepatocyte death and subsequently disease progression. We provide evidence that *Klf10* protects mouse primary hepatocytes against TNFα induced apoptosis. This is in contrast to many previous studies showing that KLF10 acts as a tumor suppressor through TGF signaling by playing an important role in induction of apoptosis^36,67^. However, all these studies that supported the pro-apoptotic role of KLF10 were conducted in cancer cells while we used primary cells. So far the role of KLF10 in humans has been only investigated in pancreatic, breast and prostate cancer cells where it has been described to inhibit proliferation and to induce apoptosis thus having a potential tumor suppressor function^40^. The role of KLF10 in human liver remains unknown and our work should stimulate studies to investigate its role in the development of NAFLDs in obese patients.

Despite a growing body of evidence supporting the relevance of circadian timing for the prevention, diagnosis and treatment of diseases, this remains mostly overlooked in the current general medical, occupational and clinical practice. Our data adds a new piece of evidence to suggest that taking into account circadian timing may be critical for both the study and the management of NAFLD. Most preclinical studies using NAFLD models are performed without any circadian timing consideration and our results suggest that this could lead to important biases in the interpretation of the data. Along the same line, as the diagnosis of NALFD patients is highly constrained by logistical and organizational considerations, its accuracy may be questioned in the light of the disease related circadian variations seen in mice if this would also hold true for humans. This remains to be evaluated, yet a rhythmicity of ALT activity has already been reported in cirrhotic patients^68^. Finally, although no approved treatment is yet available for NAFLD, future trials designed to tackle this current limitation may also be improved by using a timed delivery of the assessed drugs. This is the basis for chronotherapy which already has proven to be effective for improving the tolerability and/or efficacy of treatments targeting cancer, asthma, infectious agents or inflammation^69,70^. This innovative therapeutics approach would be particularly relevant to hepatopathy as the liver is probably the most rhythmic organ.

## Materials and methods

### Animals and study design

Animal experiment procedures were carried out in accordance with the CNRS and INSERM institutional guidelines. The local ethical committee (Comité Institutionnel d'Éthique Pour l'Animal de Laboratoire CIEPAL-AZUR, national agreement No. 28 PEA No. NCE 20-355) specifically approved this study. All the animals were housed under a 12 h light/12 h dark cycle in a temperature and humidity controlled facility. WT and *Klf10*^*-/-*^ mice in the C57BL/6j genetic background were generated by crossing of heterozygous animals. Genotyping of the mice was performed by polymerase chain reaction (PCR) of genomic DNA. For experiments, 3–6 month-old WT (Janvier-Labs), and WT / *Klf10*^*-/-*30^ male mice were fed ad libitum either a methionine- and choline-deficient diet (MCDD) or control diet (CD) for 4 weeks [ MCDD # E15653-947 ; Ctrl D # E15654-047 (SSNIFF, Soest, Germany)]. We used male mice because it has been shown that male mice are more susceptible to the progression of NAFLD in response to MCDD than female mice ^71^.Blood was sampled via retro-orbital punction and mice were sacrificed by cervical dislocation. For the circadian time series, blood sampling and animal sacrifice were performed every 6 h over 24 h at Zeitgeber (ZT) 3, ZT9, ZT15 and ZT21, ZT0 being the time where light is switched on (7.00 am). Following sacrifice, liver and kidney were sampled as described in the following section. For experiments comparing the WT and *Klf10*^*-/-*^ genotypes, blood and liver were sampled at ZT3, ZT9 and ZT21.

### Tissue sampling and preparation

Upon sampling, kidneys and pieces (50 to 100 mg) of the liver were immediately frozen in liquid nitrogen and stored at − 80 °C until analysis. Another piece of the liver (200 to 250 mg) was fixed in buffered formalin, paraffin-embedded, sectioned, and stained with Hematoxylin–Eosin. A third piece (400 to 500 mg) was used for immune cells extraction and flow cytometry analysis.

### Triglyceride extraction and quantification

Lipids were extracted from 50 to 100 mg of frozen liver tissue using the methanol:chloroform phase extraction method. Livers were first sonicated and dissolved in methanol (Sigma-Aldrich, St Quentin Fallavier, FR). Then 2 vol of CHCl_3_ were added and lysates were incubated at 4 °C overnight. Samples were rinsed with 0.05% CaCl_2_ and the organic phase was collected and evaporated using speed-vacuum centrifuge. Dry pellets were resuspended in a PBS-BSA solution (5%). TG quantification was then performed using the Triglycerides FS kit (# CQN : KS, Dia Sys) according to the manufacturer recommendations.

### Real-time quantitative PCR analysis

Real-time quantitative PCR analysis have been performed as previously described^44,72^ and described in the Supplementary Methods. Briefly, total liver RNA was extracted from 20 to 25 mg of tissue, using the RNeasy Mini Kit (74104, Qiagen, Hilden, Germany) and treated with Turbo DNA-free DNase (AM 1907, Thermo Fisher Scientific Inc., Waltham, MA, USA) according to the manufacturer’s protocol. The quantity and quality of the RNA samples were determined using the Agilent 2100 Bioanalyzer with RNA 6000 Nano Kit (#5067-1511, Agilent Technologies, Santa Clara, CA, USA). Total RNA (1 µg) was reverse transcribed with the High-Capacity cDNA Reverse Transcription Kit (Thermo Fisher Scientific Inc., Waltham, MA, USA). Real-time quantitative PCR was performed for each sample using the StepOne Plus Real-Time PCR System (Thermo Fisher Scientific Inc., Waltham, MA, USA). Gene expression was normalized to the *B2m* gene and calculated based on the comparative cycle threshold CT method (2^–ΔΔCt^). Clock genes were determined using the same methods with sybergreen detection as previously described ^72^. All primers and assays used are described in Supplementary Methods.

### Serum transaminases activity measurement

After coagulation, serum was prepared by a centrifugation 10,000 for 5 min. Determination of serum transaminases (AST/ALT) was performed using in vitro test with pyridoxal phosphate activation on Roche/Hitachi cobas c systems (ASTPM, ALTPM, cobas, Meylan, France). Roche/Hitachi cobas c systems automatically calculated the concentration of each sample.

### Immune cells staining and flow cytometry analysis

A fraction of the mouse liver (400 to 500 mg) was cut into very small pieces before incubation in RPMI 1640 medium containing 0.01% DNAse I (Roche Diagnostics) and 0.05% collagenase type IV (Sigma-Aldrich, St Quentin Fallavier, FR) for 1 h at 37 °C under agitation. Then the pieces were crushed on a 100 μm cell strainer and washed with RPMI 1640 medium. The cellular suspension was centrifuged at 630 g for 5 min at room temperature (RT). The cell pellet was resuspended and centrifuged on a density cushion of Percoll 40% and 70% at 780 g for 20 min at RT. The NPC fraction located between 40 and 70% Percoll was collected, centrifuged at 630 g for 5 min at 4 °C and resuspended in PBS containing 3% FBS, 5 mM EDTA to perform flow cytometric analysis. The cells were surface-stained with antibodies against mouse CD45 (clone 30-F11), Ly6G (clone 1A8), Ly6C (clone AL-21) purchased from BD Biosciences (Le pont de claix, FR) and eBioscience (Paris, France). Cells were acquired using a fluorescence activated cell sorting (FACS) Canto II flow cytometer, and the data were analyzed using FlowJo (BD Biosciences).

### Cellular models and treatments

Primary hepatocytes were isolated as previously described^73^ and described in Supplementary Methods. *Klf10* siRNAs (#11320001 MSS238499, Life Technologies, St Aubin, France) or control siRNA (#12935400, Life Technology, St Aubin, FR) were transfected into 200,000 (MTT) or 400,000 (western blot and flow cytometry) viable primary hepatocytes using lipofectamine RNAiMAX (#13778075, Life Technologies) according to the manufacturer’s recommendations. After 48-h transfection, cells were treated with TNFα (#315-01A Preprotech, Neuilly-sur-seine, France) (20 ng/ml) and actinomycin D (#A9415 Sigma-Aldrich, St Quentin Fallavier, FR ) (0.1 µg/ml) in a William medium containing 0.5% BSA for 12 h or 16 h as indicated.

### MTT and cell death assays

Cell survival was assessed using the MTT assay using 3-(4,5-dimethylthiazol-2-yl)-2,5-diphenyltetrazolium bromide (#M2003-1G Sigma-Aldrich, St Quentin Fallavier, FR) according to the manufacturer’s recommendations. Cell death was analyzed using a double fluorescent staining annexin-V-PE and 7-AAD according to the manufacturer’s instructions (AnnexinV-PE apoptosis detection kit I, BD Biosciences, Le pont-de-claix, France). Single cell fluorescence was analyzed by flow cytometry (BD Canto II) and FlowJo software (BD Biosciences).

### Immunoblotting

Cells were solubilized in lysis buffer (20 mM Tris, pH 7.4, 150 mM NaCl, 10 mM EDTA, 150 mM NaF, 2 mM sodium orthovanadate, 10 mM pyrophosphate, protease inhibitor cocktail, and 1% Triton X-100) for 45 min at 4 °C. Lysates were cleared (14,000 rpm, 15 min). Proteins were quantified (BCA Protein assay kit, 23225 Thermo Fisher Scientific Inc, Waltham, MA, USA.), separated by SDS-PAGE and immunoblotted as previously described^74^. The proteins were probed with anti-caspase 3 (#9662, Cell Signaling) and anti-HSP90 (#4877, Cell Signaling) antibodies at 1 µg/mL as indicated. Detection was performed using electro chemio-luminescent method (Western Lightning Plus NEL105001EA) exposure to Amersham Hyperfilm ECL (#28906837 GE life sciences, Pittsburgh, PA). Densitometry analysis was performed using Fiji software. Densitometry of the detected c-casp3 fragment was normalized by the densitometry of the detected HSP90 band of the corresponding sample.

### TUNEL assay

Liver paraffin embedded sections were deparaffinized, rehydrated and stained for 3′ hydroxyl termini of DNA double strand breaks using the Apop Tag plus Peroxidase in situ Apoptosis kit (S7101, Merck, Meyzileu, FR), and nuclei were counter-colored using hematoxylin. All the steps were performed according to the manufacturer’s instructions. The whole sections were then imaged using the Vectra Polaris digital slide scanner (CLS143455, Akoya Biosciences Inc, Marlborough, MA, USA). Image analysis were performed via IHC-based cell segmentation and TUNEL positive nuclei quantification using the HALO software (Incidia labs, Albuquerque, NM, USA). Percentage of positive nuclei was then normalized by the total analyzed surface.

### Statistical analysis

Circadian rhythmicity of time series was assessed using the non-linear regression analysis based cosinor method implemented in R as described previously^30,72^. Statistical significance was determined using a boot-strap analysis to compare confidence intervals between oscillation parameters (mean level, amplitude and phase). Significance threshold was set at p < 0.05. For Single time point data, statistical significances between two groups were determined using the non-parametric Mann–Whitney test. Differences were considered significant for p < 0.05.

## Supplementary information

Supplementary Information. (PDF 2019 kb)
