# Supplementary material for: MCD diet-induced steatohepatitis generates a diurnal rhythm of associated biomarkers and worsens liver injury in Klf10 deficient mice
Source: Sci Rep. 2020 Jul 22;10:12139. doi: 10.1038/s41598-020-69085-w (PMC7376252; doi:10.1038/s41598-020-69085-w)
Supplement: Supplementary file 1 — Supplementary Information. (PDF 2019 kb) [file 41598_2020_69085_MOESM1_ESM.pdf]

MCD Diet-induced steatohepatitis generates a diurnal rhythm of associated biomarkers and worsens liver injury of *Klf10* deficient mice

Pierre S. Leclère (1,2), Déborah Rousseau (2), Stéphanie Patouraux (3), Sophie Guérin (1), Stéphanie Bonnafous (3), Aline Gréchez-Cassiau (1), Anthony A Ruberto (1), Carmelo Luci (2), Malayannan Subramaniam (4), Albert Tran (3), Franck Delaunay (1), Philippe Gual (2)*[$], Michèle Teboul (1)*[$]

(1) Université Côte d'Azur, CNRS, INSERM, iBV, Nice, France

(2) Université Côte d'Azur, INSERM, C3M, Nice, France

(3) Université Côte d'Azur, CHU, INSERM, C3M, Nice, France

(4) Department of Biochemistry and Molecular Biology, Mayo Clinic, Rochester, MN, USA.

$ Shared last author

* Corresponding author

Philippe Gual
Philippe.gual@inserm.fr

Michèle Teboul :
Michele.TEBOUL@univ-cotedazur.fr

Supplementary Methods

Real-time quantitative PCR analysis: TaqMan gene expression assays were purchased from Thermo Fisher Scientific Inc.: *B2m (*Mm00437762_m1); *Klf10 (*Mm00449812_m1*)*; *Cidec (*Mm00617672_m1*); Pnpla2 (*Mm00503040_m1*; Tnfa (*Mm00443258_m1*); Ccl2 (*Mm00441242_m1*) ; Tgfβ1* (Mm03024053_m1) ; *Col1a1* (Mm00801666_g1) ; *Timp1* (Mm00441818_m1) ; *Klf4 (*Mm00516104_m1*); Klf6 (*Mm00516184_m1*) Klf11*(Mm00462958*). ; Il-6 (Mm00446190_m1) ; Ikb* (Mm00456849_m1).

Primers for SYBR green approach: *B2m* F: TGCTATCCAGAAAACCCCTCAA, R: GGGGTGAATTCAGTGTGAGCC; *Klf10* F: AGTGACTTTGAAGCGGTGGA, R: AAGGTGCGTTAAACAAAATGC; *Bmal1* F: CTCATTGATGCCAAGACTGG, R: GGTGGCCAGCTTTTCAAATA; *Rev-erbα*: F: AACCTCCAGTTTGTGTCAAGGT, R: GATGACGATGATGCAGAAGAAG; *Rev-erbβ:* F: TTCCAGTAGGTGGATGTTCTCA, R: CTGCTGGGGTAAAACTCATTG; *Rorc*: F: TTTTGAGGAAACCAGGCATC, R: CCACATCTCCCACATTGACTTC; *Dbp*: F: CTGAGGAACAGAAGGATGAGAAG, R: GTAGCGTGAAAGCACAGCAC.

Mouse primary hepatocytes preparation. Mouse hepatocytes were isolated with a two-step collagenase procedure. Briefly, mouse livers were perfused with HEPES buffer containing 8 g/l NaCl, 33mg/l Na2HPO4, 200 mg/l KCl and 2.38 g/l HEPES, pH 7.5, supplemented with 0.5mM EGTA for 3min at 3ml/min, then with HEPES buffer for 3 min at 3ml/min and finally with HEPES buffer supplemented with 1.5 g/l CaCl2 and 0.026% collagenase type IV (#C5138) (Sigma-Aldrich;, Saint-Quentin-Fallavier, FR) for 7 min at 3 ml/min. Livers were then carefully removed and minced in Williams' E medium (Life Technologies, St Aubin, FR) supplemented with 10% fetal bovine serum (PAA Laboratories, Velizy-Villacoublay, FR), 100 units/ml penicillin, 100 mg/ml streptomycin, 2mM L-glutamine and 0.02 UI/ml insulin (Umulin, Lilly France, Neuilly-sur-Seine, FR). The cell suspension was then filtered (100 µm) and hepatocytes were collected by centrifugation at 50 x *g* for 5 min. Viability was evaluated by trypan blue exclusion (Sigma-Aldrich, Saint-Quentin-Fallavier, FR).

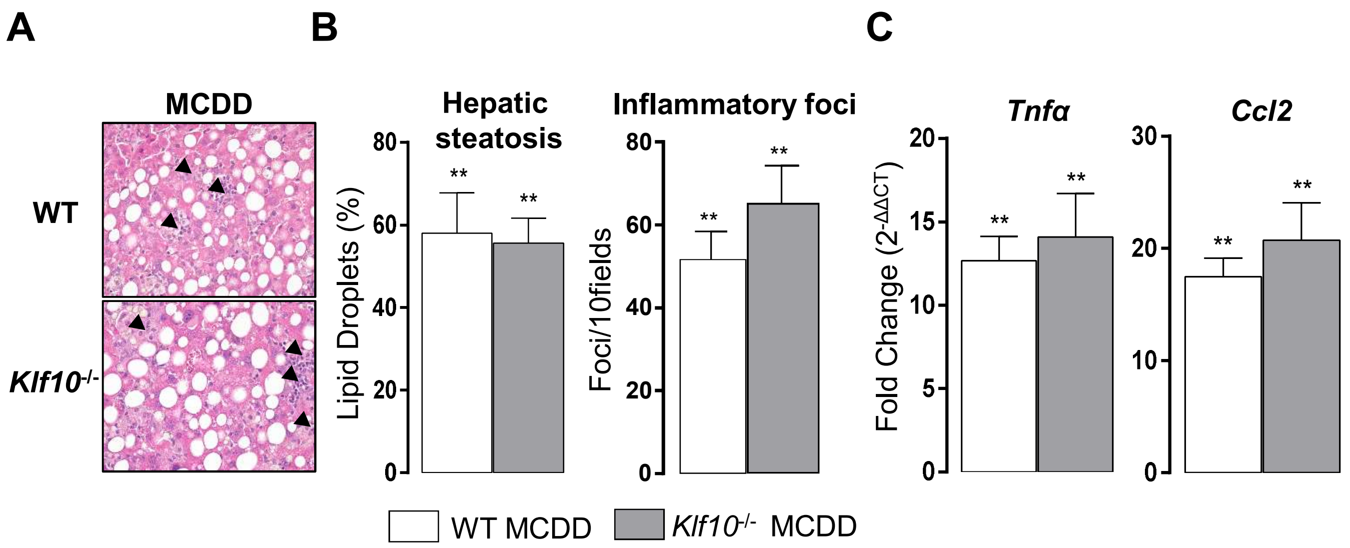

Fig. S1 *Klf10* deficiency does not impact the development of hepatic steatosis and inflammation during steatohepatitis in mice sampled at ZT9. WT and *Klf10*$^{-/-}$ male mice were fed a methionine and choline deficient diet (MCDD) for 4 weeks and sacrificed at ZT9 (n=9 mice/ group). (**A**) Representative images, showing the presence of lipid droplets and inflammatory foci (black arrows), from H&E stained liver sections of WT and *Klf10*$^{-/-}$ upon MCDD. (**B**) Quantification of hepatic steatosis and inflammatory foci from H&E stained liver sections. (**C**) Hepatic expression of *Tnfα* and *Ccl2*. All the data are expressed as mean ± SEM. The mRNA levels are normalized to *B2m* and expressed relative the CD level ZT9 (n=3). Statistical significance was tested using the Mann-Whitney test, ** $p<0.01$.

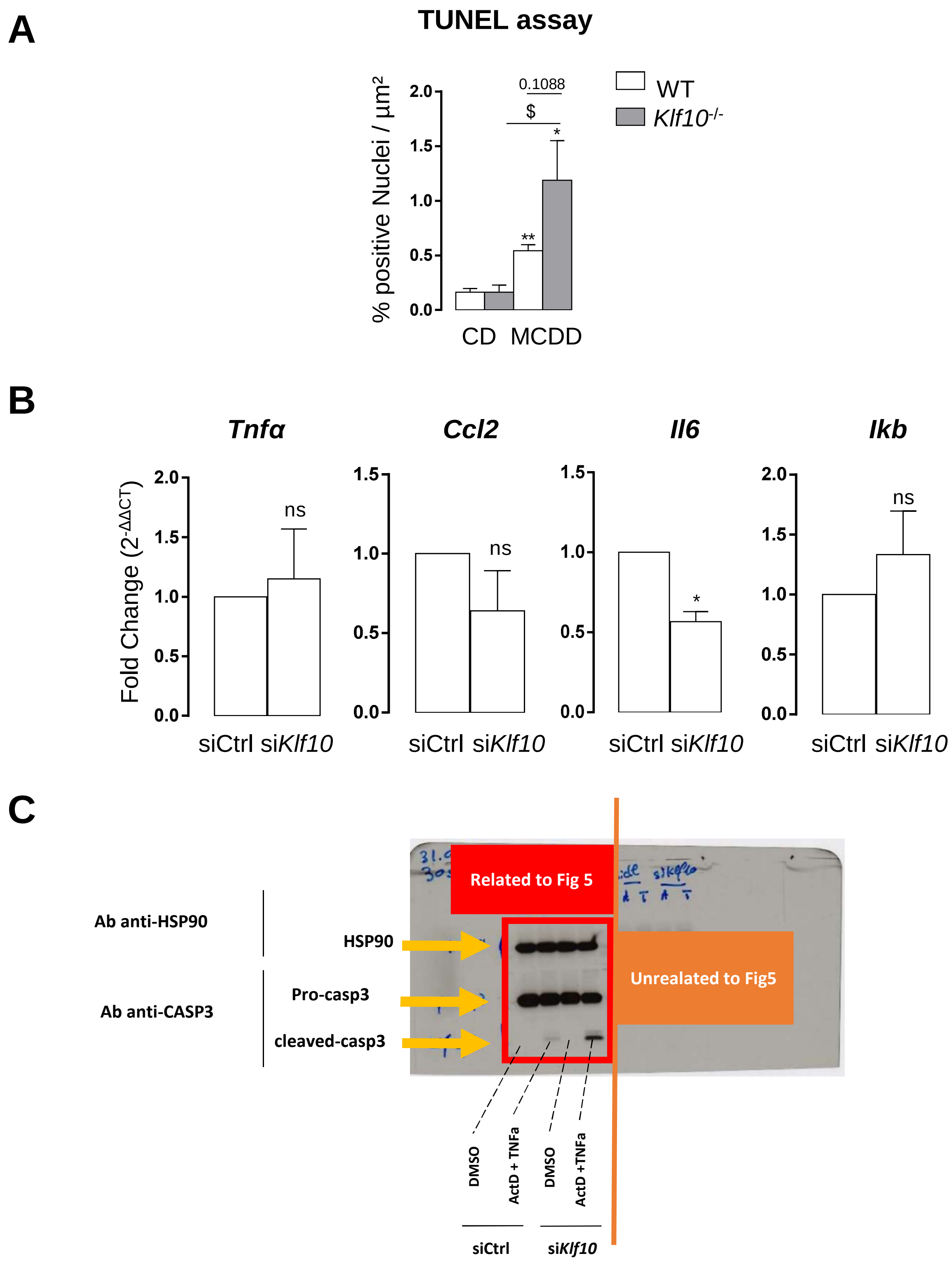

Fig. S2 *Klf10* deficient mice may display increased hepatic apoptotic cells upon MCDD challenge.
(A) quantification of the TUNEL assay performed on liver sections of WT and *Klf10*$^{-/-}$ mice fed a CD or MCDD and sacrificed at ZT3 (n=3-10 mice /group). **(B)** Mouse primary hepatocytes were transfected with control (siCtrl) or *Klf10* (si*Klf10*) siRNA. Gene expression of *Tnfα*, *Ccl2*, *Il6* and *Ikb* were evaluated. Gene expression was normalized to *B2m* and expressed as relative expression of the untreated siCtrl condition, 72h after transfection (n=4). **(C)** Corresponding uncropped image of the western blot shown in Fig. 5d. All the data are expressed as mean ± SEM. Statistical significance was tested using the Mann-Whiney test vs the control group (stars) or the indicated groups (dollar symbols), ** $p<0.01$; * and $ $p<0.05$.

Items	Diet	Mean level	Amplitude	Acrophase (ZT : min)
**Steatosis (%)**	CD	ND	ND	ND
	MCDD	12.10 (7.83 ; 16.42)*	13.19 (7.32 ; 19.25)	08:07 (06:19 ; 10:06)
**Inflammatory foci (foci / 10 fields)**	CD	ND	ND	ND
	MCDD	12.49 (7.08 ; 19.10)*	12.27 (3.72 ; 21.08)	07:38 (04:24 ; 11:08)
**Hepatic TG (mg/g of liver)**	CD	2.98 (2.47 ; 3.58)	1.59 (0.87 ; 2.35)	14.27 (12.19 ; 16.36)
	MCDD	7.35 (5.59 9.49)*	NSR	NSR
**ALT (IU/L)**	CD	20.02 (16.36 ; 25.13)	NSR	NSR
	MCDD	84.20 (74.35 ; 94.92)*	NSR	NSR
**AST (IU/L)**	CD	202 (179 ; 227)	NSR	NSR
	MCDD	620 (553 ; 689)	307 (211 ; 400)	00:57 (00:15 ; 02:00)
Pnpla2	CD	1.08 (0.93 ; 1.24)	0.43 (0.20 ; 0.64)	08:35 (06:29 ; 10:46)
	MCDD	2.02 (1.79 ; 2.30)*	0.66 (0.30 ; 1.01)	08:44 (06:22 ; 10:59)
Fsp27/Cidec	CD	1.28 (0.85 ; 1.75)	NSR	NSR
	MCDD	17.96 (14.90 ; 20.92)	7.73 (3.73 ; 11.93)	06:25 (04:04 ; 08:31)
Tnfα	CD	1.20 (1.00 ; 1.43)	NSR	NSR
	MCDD	8.73 (7.01 ; 10.78)*	5.77 (3.21 ; 8.39)	4:26 (02:19 ; 06:13)
Ccl2	CD	0.88 (0.49 ; 1.48)	NSR	NSR
	MCDD	6.73 (5.40 ; 8.29)*	4.55 (2.61 ; 6.53)	02:20 (00:32 ; 04:11)
Tgfβ1	CD	0.82 (0.72 ; 0.92)	NSR	NSR
	MCDD	2.02 (1.79 ; 2.24)*	NSR	NSR
Col1α1	CD	1.00 (0.78 ; 1.20)	NSR	NSR
	MCDD	3.63 (2.81 ; 4.49)*	1.89 (0.72 ; 3.15)	06.42 (03.44 ; 09.15)
Timp1	CD	1.11 (0.69 ; 1.57)	NSR	NSR
	MCDD	13.41 (10.05 ; 16.85)*	8.00 (3.36 ; 12.57)	06.02 (03.16 ; 08.27)

Table S1. NAFLD features cosinor analysis

Bootstrap analysis was performed to generate the 95% confidence intervals. * indicates statistical difference between CD and MCDD fed mice ($p<0.05$). NSR, not significantly rhythmic, ND, not detectable.

Gene	Diet	Mean level	Amplitude	Acrophase (ZT : min)
Bmal1	CD	0.83 (0.67 ; 1.00)	0.75 (0.52 ; 0.99)	22:20 (21:10 ; 23:35)
	MCDD	1.10 (0.96 ; 1.28)	1.21 (0.96 ; 1.46)	19:12 (18:29 ; 19:54)*
Rev-erbα	CD	0.43 (0.28 ; 0.61)	0.57 (0.33 ; 0.81)	04: 26 (02:44 ; 06:07)
	MCDD	0.36 (0.22 ; 0.54)	0.56 (0.33 ; 0.80)	03:17 (01:38 ; 04:53)
Per2	CD	7.13 (5.58 ; 8.88)	7.63 (5.31 ; 9.97)	11:36 (10:22 ; 12:49)
	MCDD	7.68 (6.91 ; 8.47)	5.85 (4.74 ; 6.95)	10:34 (09:50 ; 11:16)
Cry1	CD	2.04 (1.65 ; 2.38)	1.64 (1.18 ; 2.12)	19:23 (18:10 ; 20:39)
	MCDD	2.22 (2.09 ; 2.37)	1.88 (1.69 ; 2.07)	16:26 (16:01 ; 16:50)*
Rev-erbβ	CD	0.78 (0.64 ; 0.95)	0.55 (0.34 ; 0.78)	08:07 (06:31 ; 09:49)
	MCDD	0.47 (0.41 ; 0.54)*	0.35 (0.26 ; 0.44)	08:19 (07:11 ; 09:23)
Rorc	CD	2.66 (2.27 ; 3.15)	1.16 (0.56 ; 1.77)	15:53 (13:34 ; 18:02)
	MCDD	2.87 (2.63 ; 3.13)	2.44 (2.12 ; 2.78)*	14:35 (13:58 ; 15:10)
Dbp	CD	1.69 (1.22 ; 2.19)	2.32 (1.69 ; 3.00)	07:44 (07:41 ; 09:56)
	MCDD	0.63 (0.47 ; 0.80)*	0.88 (0.62 ; 1.14)*	8:42 (07:33 ; 09:46)
Klf10	CD	1.99 (1.40 ; 2.67)	1.50 (0.59 ; 2.41)	9:23 (6:46 ; 12:00)
	MCDD	2.50 (2.06 ; 3.00)	NSR	NSR
Klf11	CD	0.70 (0.49 ; 0.97)	0.51 (0.18 ; 0.87)	02:57 (23:54 ; 05:58)
	MCDD	0.95 (0.83 ; 1.07)	0.50 (0.34 ; 0.68)	01:31 (00:09 ; 2:52)
Klf4	CD	0.98 (0.78 ; 1.24)	NSR	NSR
	MCDD	2.64 (2.34 ; 2.94)*	NSR	NSR
Klf6	CD	0.88 (0.75 ; 1.03)	NSR	NSR
	MCDD	4.19 (3.68 ; 4.71)*	1.66 (0.97 ; 2.41)	01:17 (23:30 ; 03:06)

Table S2. Liver clock genes cosinor analysis

Bootstrap analysis was performed to generate the 95% confidence intervals. * indicates statistical difference between CD and MCDD fed mice ($p<0.05$). NSR, not significantly rhythmic.

Gene	Diet	Mean level	Amplitude	Acrophase (ZT : min)
Bmal1	CD	0.88 (0.66 ; 1.11)	0.64 (0.32 ; 0.97)	21:15 (19:09 ; 23:17)
	MCDD	1.06 (0.96 ; 1.18)	1.03 (0.87 ; 1.18)	18:19 (17:44 ; 18:53)*
Nr1d1	CD	0.68 (0.53 ; 0.85)	0.46 (0.24 ; 0.70)	7:03 (05:07 ; 08:52)
	MCDD	0.63 (0.46 ; 0.90)	0.94 (0.60 ; 1.29)	03:29 (02:04 ; 04:54)*
Per2	CD	1.41 (1.15 ; 1.66)	0.93 (0.57 ; 1.29)	13:18 (11:29 ; 14:39)
	MCDD	1.44 (0.97 ; 2.04)	1.24 (0.48 ; 2.02)	08:45 (06:02 ; 11:18)*
Cry1	CD	4.11 (3.24 ; 4.97)	3.77 (2.58 ; 4.93)	16:19 (14:59 ; 17:37)
	MCDD	8.68 (5.67 ; 3.22)*	8.28 (3.65 ; 4.06)	10:38 (07:44 ; 13:29)*
Nr1d2	CD	0.88 (0.77 ; 1.00)	0.46 (0.29 ; 0.62)	08:59 (07:38 ; 10:16)
	MCDD	1.09 (0.67 ; 1.68)	1.23 (0.51 ; 1.93)	05:52 (03:00 ; 08:15)
Rorc	CD	2.39 (2.46 ; 3.51)	2.54 (1.85 ; 3.24)	16:30 (15:15 ; 17:36)
	MCDD	3.38 (3.17 ; 4.76)	2.2 (1.11 ; 3.38)	13:07 (11:03 ; 15:28)
Dbp	CD	1.77 (51.38 ; 2.15)	2.01 (1.44 ; 2.60)	09:41 (08:40 ; 10:43)
	MCDD	2.22 (1.88 ; 2.57)	2.68 (2.20; 3.19)	05:49 (05:08 ; 06:29)*
Klf10	CD	2.02 (1.71 ; 2.35)	1.18 (0.77 ; 1.59)	14:41 (13: 07 ; 16:22)
	MCDD	2.15 (1.71 ; 2.67)	NSR	NSR
Klf11	CD	0.68 (0.56 ; 0.83)	0.33 (0.16 ; 0.51)	03:15 (00:36 ; 05:42)
	MCDD	0.85 (0.69; 1.02)	0.60 (0.37; 0.83)	03:34 (01: 55; 05:03)

Table S3. Kidney clock genes cosinor analysis

Bootstrap analysis was performed to generate the 95% confidence intervals. * indicates statistical difference between CD and MCDD fed mice ($p<0.05$). NSR, not significantly rhythmic.

.
